# Operational Research on Operational Research: Assessing the Impact of the Structured Operational Research and Training Initiative on Tackling Antimicrobial Resistance in Ghana

**DOI:** 10.3390/tropicalmed10110312

**Published:** 2025-10-31

**Authors:** Rony Zachariah, Pruthu Thekkur, Fiona Braka, Nienke Bruinsma, Anthony D. Harries, Christine M. Halleux, Kwame Ohene Buabeng

**Affiliations:** 1 UNICEF/UNDP/World Bank/WHO Special Programme on Research and Training in Tropical Diseases (TDR), 20, Avenue Appia, 27, 1211 Geneva, Switzerland; halleuxc@who.int; 2 International Union Against Tuberculosis and Lung Disease, 2 Rue Jean Lantier, 75001 Paris, France; pruthu.tk@theunion.org (P.T.); adharries@theunion.org (A.D.H.); 3 Ghana Country Office, World Health Organization, 12 Agbaamo St, Accra P.O. Box 142, Ghana; brakaf@who.int; 4 Antimicrobial Resistance Department, World Health Organization, 20, Avenue Appia, 27, 1211 Geneva, Switzerland; bruinsman@who.int; 5 Department of Clinical Research, Faculty of Infectious and Tropical Diseases, London School of Hygiene and Tropical Medicine, Keppel Street, London WC1E 7HT, UK; 6 National Antimicrobial Resistance Policy Platform, Accra P.O. Box 44, Ghana; kbuabeng@uhas.edu.gh; 7 Department of Pharmacy Practice, School of Pharmacy, University of Health and Allied Sciences, Ho P.O. Box 31, Ghana

## 1. Introduction

*“If research is to have impact and improve outcomes, it must be locally relevant and the findings actionable to shape policy and/or practice. SORT IT is well designed and invaluable for this purpose.”*

*Dr Sartie Kenneh, Chief Medical Officer, Ministry of Health and Sanitation, Sierra Leone*

Some years ago, Ian Chalmers and Paul Glasziou published a landmark paper in The Lancet titled “*Avoidable Waste in the Production and Reporting of Research Evidence.*” They estimated that as much as 85% of research investment may be wasted because of flaws in the way evidence is produced and reported [1]. As a result, many studies remain incomplete or fail to translate into policies or practices that could improve health [1]. This waste occurs in four areas: the relevance of research questions, study design and methods, efficient conduct and accessibility, and reporting and publication [1]. There is additional waste that occurs beyond the publication milestone due to restricted access, poor translation, inefficient search systems, and other problems with the dissemination and use of research [2].

With an estimated global health research spending of approximately USD 240 billion annually, and assuming the 85% research waste rate holds, this could imply that as much as USD 204 billion may be wasted each year [3,4]. This is a staggering figure. To put this into perspective, if research were a transport or freight service, more than half the cargo would be lost in transit, and much of the remainder would arrive broken, defective, or flawed upon arrival. In other words, the majority of the investment fails to meet the needs or expectations of the end user. At best, this represents gross inefficiency; at worst, it is fraudulent—delivering only a fraction of the promised goods.

The COVID-19 pandemic further highlighted the global scale of this problem. The World Health Organization’s (WHO) International Clinical Trials Registry Platform (ICTRP) has logged over 20,000 COVID-19 trial registrations; however, fewer than 10% are estimated to have meaningfully informed decision-making. Existing clinical trial frameworks and guidance proved insufficient to prevent this waste [5].

To ensure “value for money”, accountability is key. Yet in practice, researchers and institutions rarely track the impact of their work beyond the publication milestone [6,7].

The Structured Operational Research and Training Initiative (SORT IT), led by TDR with partners from Non-Governmental Organizations, academia, and Ministries of Health, addresses this gap by embedding assessment of the influence of research on policy and practice [6,8,9]. In this editorial, we reflect on the legacy of SORT IT and how it has translated operational research into tangible health gains in Ghana, where studies were conducted to evaluate the impact of earlier research.

## 2. The SORT IT Model in Tackling Antimicrobial Resistance (AMR) and Its Legacy of Success in Asia, Africa, and the Americas (2019–2025)

Building operational research capacity is very much needed to address global public health challenges such as AMR. An estimated 1.27 million deaths each year are directly attributable to AMR, and 4.95 million deaths are associated with it [10]. This places AMR over HIV and tuberculosis as one of the world’s most important public health threats. Operational research is key to providing a roadmap that generates evidence to inform national responses and drive impactful actions on the ground.

**Figure 1:**
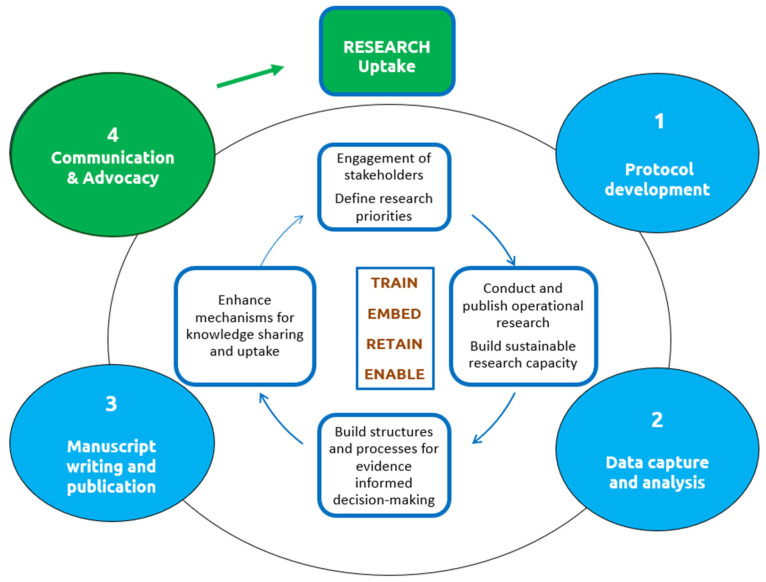
The Structured Operational Research and Training Initiative cycle and modules (numbered 1–4) conducted over 10–12 months.

The SORT IT cycle, which blends research training with real-world implementation, utilizes clear milestones, performance goals, and impact tracking, as shown in Figure 1. Over a period of 10 to 12 months, participants receive expert mentoring and complete four hands-on modules focused on research capacity building, practical skills, and producing clear outputs, such as scientific publications, plain-language policy briefs, and short PowerPoint presentations for key stakeholders and decision-makers [8].

TDR’s latest initiative, which was supported by the Department of Health and Social Care (DHSC) of the United Kingdom across seven countries globally in Africa (Ghana, Sierra Leone, Uganda), Asia (Nepal, Myanmar), and Latin America (Colombia, Ecuador), was focused on tackling AMR [11]. Altogether, surveys showed that 75 of 77 studies were completed and 79% influenced policy or informed practice at national (34%), regional (10%), or health facility (56%) levels. The program also strengthened research leadership; 92% of trainees applied their skills to address AMR; 64% conducted new studies, and 36% became mentors, building a pipeline of operational researchers. Half of those trained also applied their skills to addressing emerging infections, such as COVID-19 and Mpox, underscoring their role in strengthening health systems worldwide [12,13].

## 3. SORT IT’s Impact in Ghana: A Three-Year Cycle for Change

This Special Issue on antimicrobial resistance (AMR) in Ghana showcases results from “operational research” in a rigorous manner, documenting SORT IT’s impact three years after project completion [14]. SORT IT’s monitoring framework operates on a three-year cycle to ensure continuous improvement and measurable outcomes.

- Year 1: Conduct baseline studies and advocate findings to decision-makers.
- Year 2: Provide technical support for the implementation of findings.
- Year 3: Conduct follow-up studies to assess the impact of the implemented research.

Impact assessment is part of SORT IT’s performance targets and has been described previously [6,9]. In brief, approximately 12–15 months after study completion, a survey instrument is sent to principal investigators to determine whether the findings have influenced policy or practice. This is followed by qualitative discussions with investigators and key stakeholders, cross-verifying reported changes with documentation of new tools, strategies, or interventions. Enabling factors for change are also recorded as part of continuous learning.

**Figure 2:**
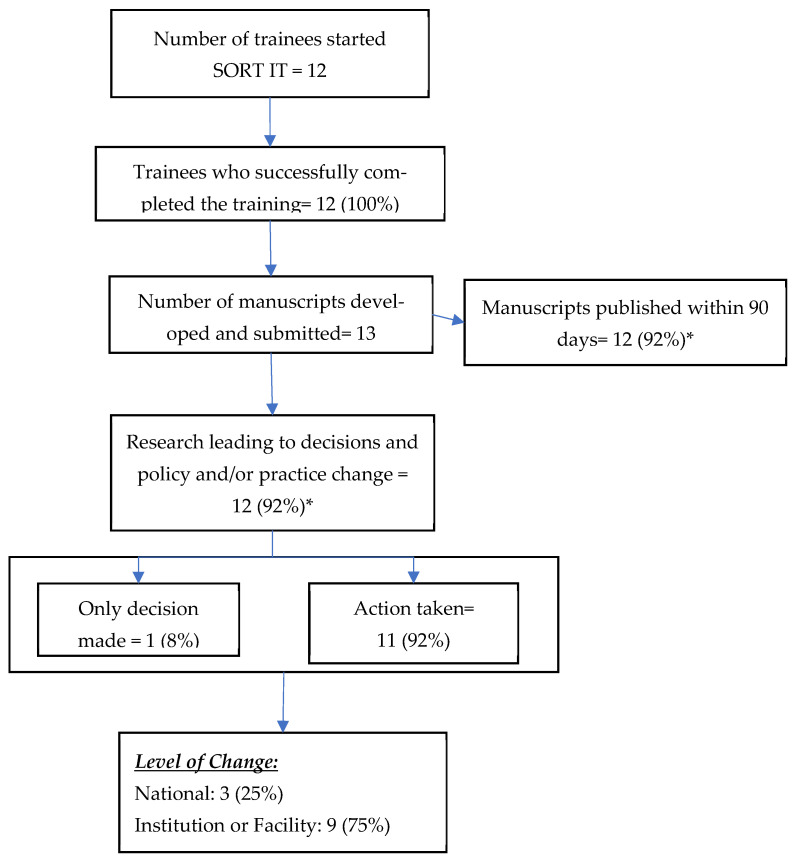
Research outputs and their impact on policy and/or practice from the Structured Operational Research and Training Initiative (SORT IT) on tackling antimicrobial resistance in Ghana, 2023–2025 (N = 13). * Percentage calculated with the total number of manuscripts as the denominator.

The flowchart (Figure 2) summarizes the journey of 13 research studies in Ghana—from initiation to publication and eventual impact. There were 12 trainees, all of 12 successfully completed the SORT IT course and produced 13 manuscripts (one trainee produced two manuscripts). An online survey conducted in 2023 among the principal investigators of 12 (92%) of the 13 manuscripts published reported that their operational research had an impact on policy and/or practice within 12 months of completing the course.

Key enabling factors, as reported by research teams, are shown in Table 1 below. These included: alignment with national priorities, early engagement of end-users, embedding capacity in national institutions for long-term sustainability, inclusive co-authorship, high-quality and timely publication, effective dissemination and communication, and hands-on mentorship with follow-up to impact. Modest funding and support for these activities were channeled to the AMR national committee through the WHO country office.

**In conclusion,** the evidence from Ghana shows that when research and capacity building are focused on national priorities, involve leaders in policy decisions at both institutional and national levels, and are backed by strong mentorship and accountability, they can result in high completion rates, and the outcomes extend beyond publications to real-world change. As global health systems confront threats like AMR and other outbreaks and beyond, the Ghana experience underscores a simple truth: research can deliver value and translate into action.

## Figures and Tables

**Table 1:** Enabling Factors for Policy and Practice Change: Insights from Operational Research in Ghana (2023–2025).

Strategic Area	Enabling Factor
*Align research with national priorities.*	All studies were vetted by the National AMR Committee to ensure relevance. The principle was clear: “*national research, with national ownership, for national solutions.*”
*Engage end-users early and continuously.*	Key decision-makers were involved throughout—from conception to publication—building shared ownership and accountability.
*Ensure timely, high-quality publications and inclusive co-authorship*	Decision-makers valued credible evidence delivered on time for use in policy and practice, with their direct involvement.
*Disseminate and communicate effectively.*	Scientific papers were translated into plain-language summaries tailored to various audiences, including community members. “*What matters is not what we say, but what they hear.*”
*Build and sustain a critical pool of researchers.*	Collaboration between public health programs and academia pooled expertise and resources, creating a pipeline of trained researchers who mentor others and renew leadership.
*Provide sustained support and follow-up.*	Continued engagement by the World Health Organization office in Ghana and the SORT IT team ensured progress in tracking, funding, and problem-solving. “*What gets measured gets done*”.
